# Association of *β*-Blocker Therapy at Discharge with Clinical Outcomes after Acute Coronary Syndrome in Patients without Heart Failure

**DOI:** 10.1155/2020/4351469

**Published:** 2020-04-24

**Authors:** Yan Chen, Xiao-fang Tang, Run-lin Gao, Yue-jin Yang, Bo Xu, Jin-qing Yuan

**Affiliations:** Department of Cardiology, The State Key Laboratory of Cardiovascular Disease, Fuwai Hospital, National Center for Cardiovascular Diseases, Chinese Academy of Medical Sciences and Peking Union Medical College, Beijing, China

## Abstract

**Aim:**

To evaluate the clinical impact of *β*-blocker in patients with adequate left ventricular ejection function (LVEF) who underwent percutaneous coronary intervention (PCI) for acute coronary syndrome (ACS).

**Methods:**

A total of 10,724 consecutive patients who underwent PCI throughout 2013 were prospectively enrolled in the study. Among these, we analyzed 5,631 ACS patients who were discharged with LVEF ≥ 40%. Patients were then compared according to the *β*-blocker prescription at discharge.

**Results:**

During a 2-year follow-up, no significant association was observed of *β*-blocker use with all-cause mortality (with *β*-blockers 47/5,043 (0.9%) *vs.* without *β*-blocker use 8/588 (1.4%); hazard ratio (HR) 0.762, 95% confidence interval 0.36 to 1.64; *P* = 0.485), cardiac death, myocardial infarction (MI), or major adverse cardiovascular and cerebrovascular events. Subgroup analysis demonstrated that the *β*-blocker use at discharge reduced the 2-year mortality in patients with unstable angina (UA) (HR 0.42, 95% CI 0.19 to 0.94, *P* = 0.034). Landmark analysis at 1 year showed that patients with UA who were discharged with *β*-blockers had lower mortality (HR 0.17, 95% CI 0.04-0.65, *P* = 0.010) and cardiac death (HR 0.12, 95% CI 0.01-0.99, *P* = 0.049) than those discharged without *β*-blockers. However, the benefit was lost beyond 1 year. No differences in outcomes were recorded in the AMI or overall population.

**Conclusions:**

We present that *β-*blocker significantly lowers the rate of all-cause death up to 1 year, in UA patients who have undergone PCI and have adequate LVEF. Its role in patients with AMI also deserves further exploration.

## 1. Introduction

The wide application of *β*-blockers in coronary artery disease patients is partially supported by the general belief that they can reduce cardiac events. American guidelines recommend oral treatment with *β*-blockers during the hospital stay in acute coronary syndrome (ACS) patients without contraindications; the oral treatment should continue even after hospital discharge regardless of the presence of left ventricular (LV) dysfunction (class I, level of evidence B). However, European guidelines have a class IIa indication for patients with adequate LV function [1–5]. These recommendations are mainly drawn from studies conducted in the prereperfusion era [6] or studies of heart failure (HF) [7] patients. Besides, mixed results have been reported on the clinical benefit of *β*-blocker in patients without HF and ACS patients who have undergone percutaneous coronary intervention (PCI) [8–11]. In as much as the use of *β-*blocker reduced mortality before the reperfusion era, a recent meta-analysis in an ACS population revealed that it was no longer the case in the modern era [11]. Furthermore, studies showing that Asians are more susceptible to the adverse effects of *β*-blockers thereby offsetting its clinical benefits in the Asian population have been published [12]. There is scant data on *β*-blocker therapy among the Asian population who underwent PCI for ACS and had mild recessive or normal LV function.

In this study, we sought to examine the association between *β*-blocker therapy at discharge and long-term clinical outcomes in ACS patients who underwent PCI with adequate LV function, from a single center of China.

## 2. Materials and Methods

### 2.1. Study Population

We consecutively enrolled 10,724 patients treated with stent implantation in Fuwai Hospital for coronary artery disease, between January and December 2013, and obtained their baseline data from the medical records.

**Figure 1:**
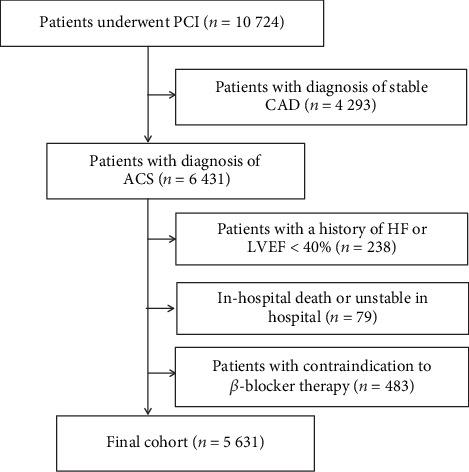
Flow diagram describing the study population. ACS: acute coronary syndrome; CAD: coronary artery disease; HF: heart failure; LVEF: left ventricular ejection fraction; PCI: percutaneous coronary intervention.

Patients with a diagnosis of ACS at admission and underwent PCI and those above 18 years formed the inclusion criteria. However, exclusion criteria included (1) patients with a history of heart failure (HF) or left ventricular ejection fraction (LVEF) < 40%, (2) in-hospital death or unstable in the hospital, (3) patients missing *β*-blocker information, and (4) patients with contraindication to *β*-blocker therapy such as hypotension (systolic blood pressure < 90 mmHg), significant bradycardia, or active asthma. Eventually, we included 5,631 patients in the study (Figure 1), and they were divided into two groups based on whether they used *β*-blockers at discharge or not.

We complied with the Declaration of Helsinki and obtained approval from the Institutional Review Board of Fuwai Hospital. In addition, all participants submitted written informed consent before the intervention.

### 2.2. Treatment and Intervention

Coronary interventions were performed by experienced cardiologists according to standard guidelines [13], and the treatment strategy, i.e., PCI and stent type, was left to the discretion of the operators. Patients received a loading dose of aspirin (300 mg) and clopidogrel (300 mg) orally and continued the dual antiplatelet therapy (aspirin (100 mg/day) and clopidogrel (75 mg/day)) for at least 12 months. Although unfractionated heparin (100 U/kg) was applied in all patients for anticoagulation during the procedure, glycoprotein IIb/IIIa antagonists were also administrated on a necessity basis. Standard secondary prevention for CAD was prescribed according to established guidelines [14].

### 2.3. Definitions and Outcomes

The follow-up was prespecified to occur after 1, 6, 12, and 24 months. The primary outcome was all-cause death, defined as all incident death that could be attributed to a cardiac or noncardiac etiology, while secondary outcomes included cardiac death, recurrent MI, and major adverse cardiovascular and cerebrovascular events (MACCE). Death that could not be attributed to a noncardiac etiology was considered a cardiac death. MI was defined by the third universal definition of myocardial infarction [15], while MACCE was defined as the composite of all-cause death, nonfatal MI, unplanned target vessel revascularization, stent thrombosis, and stroke during the follow-up. Unplanned target vessel revascularization was defined as repeat percutaneous intervention or surgical bypass of any segment of the target vessel for ischemic symptoms and event-driven. Stent thrombosis was defined according to the Academic Research Consortium, including definite and probable in the analysis.

All endpoints were adjudicated centrally by 2 independent cardiologists, and disagreement was resolved by consensus.

### 2.4. Statistical Analysis

Continuous variables are presented as the mean ± SD or median (25th and 75th percentiles) when appropriate and were compared by Student's *t*-test or the Mann-Whitney *U* test. Categorical variables are presented as frequency (percentage) and were compared using the *chi-squared* test or Fisher's exact test. Survival curves were constructed using the Kaplan-Meier method and compared using the log-rank test. To estimate the hazard ratio (HR) and the 95% confidence interval (CI) of *β*-blocker therapy and adverse event risk, we performed Cox proportional regression analysis. Variables with a *P* value < 0.05 in the univariate Cox proportional hazard model were included for further multivariate analysis (details in Table 1). Subgroup analysis was conducted with the covariates of clinical presentation (AMI *vs.* unstable angina (UA)). Additionally, we conducted the landmark analysis to assess outcomes at 1 year and between 1 and 2 years in overall and subgroups.

Significance was set at *P* < 0.05, and all analyses were performed using *SPSS 22.0* (IBM Corp., USA).

## 3. Results

### 3.1. Baseline and Procedure Characteristics

Out of the 5,631 patients discharged alive with LVEF ≥ 40% and without HF, 5,043 (89.56%) received *β*-blocker treatment at discharge. The baseline characteristics of patients are presented in Table 2. In particular, patients with *β*-blocker treatment at discharge were mainly younger, females, and nonsmokers. In addition, a larger proportion of those discharged with *β*-blocker had diabetes and dyslipidemia, presenting with AMI. Patients with *β*-blocker therapy at discharge had more complicated lesions and a higher SYNTAX score (SS). However, they recorded significantly lower residual SS (rSS) after the intervention, compared with those without *β*-blockers at discharge.

### 3.2. Two-Year Clinical Outcomes in the Overall Population

**Figure 2:**
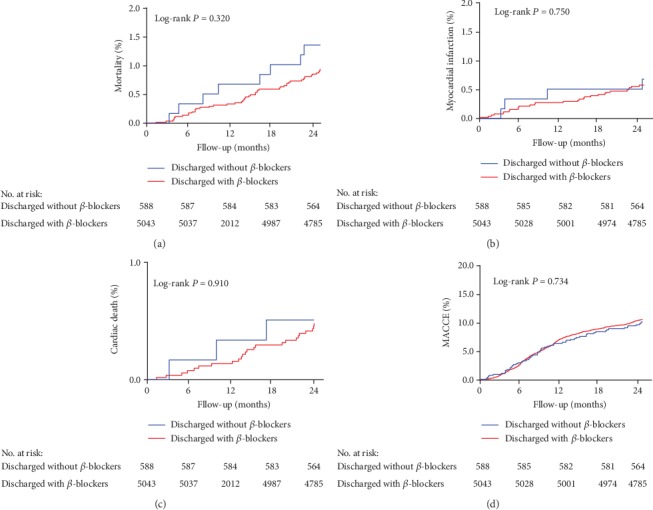
Kaplan-Meier curve for all-cause death (a), cardiac death (b), myocardial infarction (c), and MACCE (d) in the overall population. MACCE: major adverse cardiovascular and cerebrovascular events.

5,603 (99.5%) patients had complete two-year follow-up information as summarized in Table 3. Table 1 presents the landmark analysis of events occurring within and after 1 year, while Kaplan-Meier curves of the overall population for time to all-cause death, cardiac death, MI, and MACCE are shown in Figure 2. At the 2-year follow-up, the incidences of all-cause death (HR 0.69, 95% CI 0.32 to 1.47, *P* = 0.336), cardiac death (*P* = 0.925), MI (*P* = 0.338), or MACCE (*P* = 0.614) did not differ significantly between the two groups. Multivariate Cox proportional regression analysis revealed that age was an independent risk factor, while clopidogrel use was a protective factor for 2-year all-cause death. Moreover, age, prior PCI, and rSS > 8 were independent risk factors for 2-year cardiac death, while LVEF was a protective factor. Prior coronary artery bypass grafting and rSS > 8 were however independent risk factors for 2-year nonfatal MI. Additionally, left ascending artery lesion, GPIIb/IIIa use, and rSS > 8 were independent risk factors for 2-year MACCE. Rates of clinical outcomes within and after 1 year were similar between the two groups as revealed by the landmark analysis.

### 3.3. Subgroup Analysis

**Figure 3:**
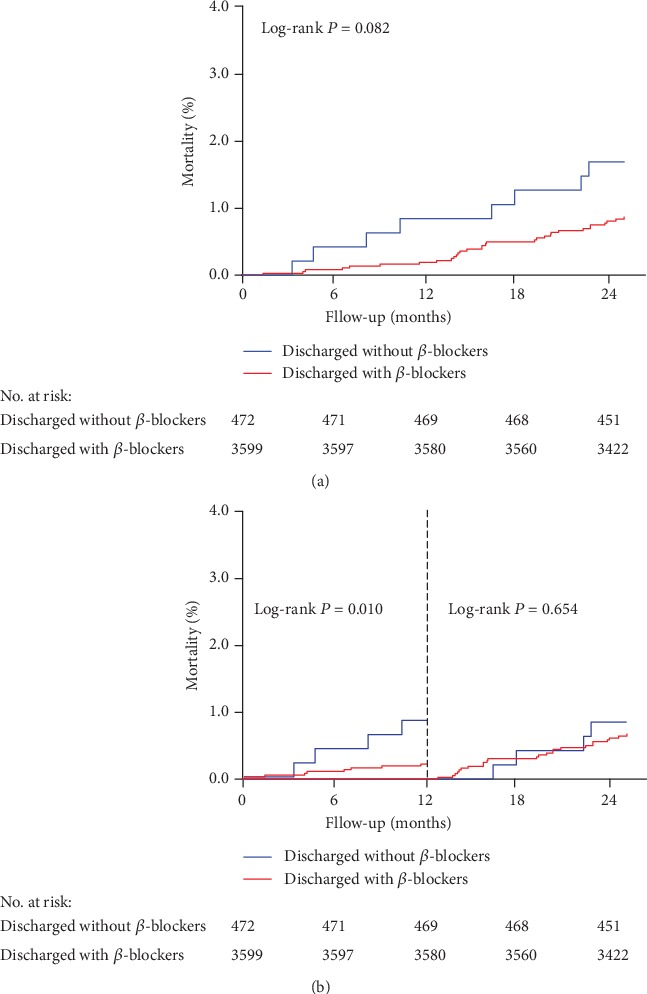
All-cause death in patients with unstable angina: (a) incidence of all-cause death in two groups; (b) landmark analysis discriminating between death occurring before and after 1-year follow-up.

Table 2 shows the baseline and procedure characteristics of subgroups, while Tables 1 and 3 summarize the 2-year clinical outcomes and landmark analyses. Patients discharged with *β*-blocker and manifesting unstable angina (UA) recorded significantly lower 2-year mortality compared with those discharged without *β*-blocker (HR 0.42, 95% CI 0.19-0.94, *P* = 0.034). Together with *β*-blocker use, clopidogrel use served as a protective factor for all-cause death. Besides, landmark analysis showed that the use of a *β*-blocker was effective on all-cause death after the first year of follow-up (Figure 3). However, the risk of all-cause death beyond 1 year was similar between the two groups. Although not statistically significant (HR 0.36, 95% CI 0.10-1.32, *P* = 0.123), we observed a decline in 2-year cardiac deaths among patients discharged with *β*-blockers. Landmark analysis also revealed a protective effect of *β*-blocker use on 1-year cardiac death, but not beyond 1 year (Table 1). There was no impact of *β*-blocker use at discharge on 2-year MI or MACCE. In the UA subpopulation, clopidogrel use was a protective factor for 2-year cardiac death. In addition, the prior coronary artery bypass graft and rSS > 8 were independent risk factors for 2-year nonfatal MI, while left ascending artery lesion and rSS > 8 were independent risk factors for 2-year MACCE.

In the AMI subpopulation, we did not observe any association of *β*-blocker use at discharge, with clinical outcomes, shown either within or after a 1-year follow-up. However, in patients with AMI, stroke and left main lesion were significantly related to 2-year all-cause death; hence, we considered prior MI and left main lesion to be independent risk factors for 2-year cardiac death. Peripheral vascular disease, left main lesion, and rSS > 8 were independent risk factors for 2-year nonfatal MI, while left ascending artery lesion and rSS > 8 were independent risk factors for 2-year MACCE.

## 4. Discussion

In the present study, we investigated the association of *β*-blocker therapy with clinical outcomes in real-life patients using data from a large, prospective, single-center series in China. Moreover, among ACS patients who underwent PCI with normal or mildly reduced LVEF, the use of *β*-blockers at discharge did not correlate with a lower risk of clinical outcomes up to 2 years. However, in the subgroup analysis of the UA population, a reduction of mortality with *β*-blocker therapy at discharge was observed, though the superiority was only significant within the first year, but not after a 2-year follow-up.

*β*-Blockers are accepted as the standard care for coronary heart disease, especially in MI patients. As mentioned before, there is a divergence between international guidelines in their recommendations for the use of *β*-blockers in ACS patients without HF, or LV dysfunction [1–4, 16]. Besides, all the recommendations relying on evidence from the prerevascularization era [6] and expert opinion (level C) only in patients with normal LV function with NSTE-ACS [7]. Despite its benefits on hard clinical outcomes in ACS patients with HF and reduced LV function being evident in the pre- and revascularization eras [9], the use of *β*-blockers remains controversial in patients undergoing PCI with adequate LV function [9, 17, 18]. Studies on the Asian population have reported inconsistent results [8, 19–21], with scant data focused on the relatively low-risk patients with normal or mild recessive LVEF. In Li et al.'s Chinese population data, *β*-blocker use significantly lowered the risk of all-cause death in ACS patients who underwent PCI. However, the relatively low-risk population without HF or LV dysfunction was not explored [19]. In Nakatani et al.'s analysis in Japanese, *β*-blocker therapy at discharge had beneficial effects for high-risk patients only [21].

This study, therefore, sought to clarify this controversy. After full adjustment of potential confounding factors, we demonstrated that *β*-blocker therapy at discharge was associated with a significant reduction in 2-year mortality in patients who underwent PCI for UA and had adequate LVEF. Landmark analysis demonstrated the benefit of *β*-blocker use on all-cause death and cardiac death at a 1-year follow-up, but not beyond that period. Since we were uncertain of the duration of *β*-blocker use after discharge, our results should be interpreted with some caution.

In the era of PCI and modern medical therapy, it is true that the clinical outcomes of relatively low-risk patients, with normal or mild recessive LV function after ACS, would be improved. However, recurrent myocardial ischemia, tachyarrhythmia, and adrenergic activation remain serious problems for these patients. Since there is no other optimal substitute for *β*-blockers in controlling these problems effectively [22], our results supported the *β*-blocker use in relatively low-risk patients who underwent PCI for UA and had adequate LVEF.

Patients with myocardial damage from AMI may have higher levels of sympathetic tone and circulating catecholamine than those with UA, hence more likely to benefit from *β*-blocker therapy. However, in the present study, we did not observe any significant impact of *β*-blocker use on clinical outcomes in the AMI population. This could be attributed to the sample size and relatively low event rates encountered in the AMI subpopulation. It is therefore not accurate to conclude from this study alone that the drug is ineffective in patients with AMI.

Beyond the medicine, the present study also revealed the importance of interventional therapy for the prognosis of the ACS population. Besides, incomplete revascularization (rSS > 8) was shown to be an independent risk factor for MI and MACCE. This was true for the overall AMI and UA populations, respectively.

This study, however, has the following limitations. First, it is an observational study; hence, conventional limitations for such apply. Secondly, the present study was conducted in a Chinese population with relatively low incidences of cardiac events. Despite the total population of 5,631 cases, only 588 of the patients did not use *β*-blockers, which is a small sample size. Thirdly, unmeasured confounders may have led to biased results. Finally, the study lacks data on specific *β*-blockers and doses, and as mentioned before, we are uncertain of the duration that *β*-blockers were administered after discharge. Large-scale, prospective, randomized controlled trials should be conducted to clarify the effects of long-term *β*-blocker therapy in ACS patients who have undergone PCI with adequate LVEF.

## 5. Conclusion

Our findings are in agreement that *β-*blocker significantly lowers the rate of all-cause death up to 1 year, in UA patients who have undergone PCI and have adequate LVEF. Its role in patients with AMI also deserves further exploration.

## Figures and Tables

**Table 1:** Landmark analyses in 2-year clinical outcomes.

	Overall population				AMI subpopulation				UA subpopulation			
	Discharged with *β*-blockers (*n* = 5,043)	Discharged without *β*-blockers (*n* = 588)	HR (95% CI)	*P* value	Discharged with *β*-blockers (*n* = 1,444)	Discharged without *β*-blockers (*n* = 116)	HR (95% CI)	*P* value	Discharged with *β*-blockers (*n* = 3,599)	Discharged without *β*-blockers (*n* = 472)	HR (95% CI)	*P* value
0-2 years												
Death	47 (0.9)	8 (1.4)	0.69 (0.32-1.47)	0.336	16 (1.1)	0 (0.0)	NA	NA	31 (0.9)	8 (1.7)	0.42 (0.19-0.94)	0.034
Cardiac death	24 (0.5)	3 (0.5)	0.94 (028-3.18)	0.925	10 (0.7)	0 (0.0)	NA	NA	14 (0.4)	3 (0.6)	0.36 (0.10-1.32)	0.123
MI	29 (0.6)	5 (0.9)	0.63 (0.24-1.63)	0.338	8 (0.6)	2 (1.7)	0.32 (0.07-1.54)	0.156	21 (0.6)	3 (0.6)	0.84 (0.25-2.81)	0.772
MACCE	538 (10.7)	61 (10.4)	0.93 (0.72-1.22)	0.614	155 (10.7)	12 (10.3)	1.02 (0.57-1.85)	0.935	383 (10.6)	49 (10.4)	0.91 (0.67-1.22)	0.517
0-1 year												
Death	17 (0.3)	4 (0.7)	0.55 (0.18-1.73)	0.316	10 (0.7)	0 (0.0)	NA	NA	7 (0.2)	4 (0.8)	0.17 (0.04-0.65)	0.010
Cardiac death	7 (0.1)	2 (0.3)	0.61 (0.11-3.26)	0.562	5 (0.3)	0 (0.0)	NA	NA	2 (0.1)	2 (0.4)	0.12 (0.01-0.99)	0.049
MI	14 (0.3)	4 (0.7)	0.40 (0.13-1.22)	0.106	3 (0.2)	1 (0.9)	0.32 (0.03-3.07)	0.322	11 (0.3)	3 (0.6)	0.69 (0.15-3.14)	0.633
MACCE	354 (7.0)	39 (6.6)	0.94 (0.68-1.32)	0.749	107 (7.4)	11 (9.5)	0.77 (0.41-1.44)	0.409	247 (6.9)	28 (5.9)	1.01 (0.68-1.50)	0.954
*>*1 year-2 years												
Death	30 (0.6)	4 (0.7)	0.80 (0.28-2.30)	0.677	6 (0.4)	0 (0.0)	NA	NA	24 (0.67)	4 (0.85)	0.66 (0.22-1.95)	0.455
Cardiac death	17 (0.3)	1 (0.2)	1.68 (0.22-12.80)	0.614	5 (0.3)	0 (0.0)	NA	NA	12 (0.3)	1 (0.2)	0.84 (0.11-6.80)	0.874
MI	15 (0.3)	1 (0.2)	1.56 (0.21-11.80)	0.669	5 (0.4)	1 (0.9)	0.35 (0.04-3.03)	0.341	10 (0.28)	0 (0.00)	NA	NA
MACCE	184 (3.9)	22 (4.0)	0.91 (0.58-1.42)	0.684	48 (3.6)	1 (1.0)	3.88 (0.53-28.16)	0.181	136 (4.1)	21 (4.7)	0.76 (0.48-1.21)	0.253

Values are presented as *n* (%). Variables with a *P* value < 0.05 in the univariate Cox proportional hazard model were included. In the overall population, for all-cause death, the variables, namely, age, stroke, COPD, prior PCI, CCr < 60 mL/min, LVEF, heart rate, rSS, and clopidogrel use, were adjusted. For cardiac death, the variables, namely, age, prior MI, prior PCI, prior CABG, CCr < 60 mL/min, LVEF, heart rate, rSS, and clopidogrel use, were adjusted. for MI, the variables, namely, CCr < 60 mL/min, prior CABG, and rSS, were adjusted. For MACCE, the variables, namely, diabetes, stroke, prior MI, prior CABG, LVEF, rSS, LAD lesion, and GPIIb/IIIa inhibitor use, were adjusted. In the UA subpopulation, variables of age, COPD, LVEF, and clopidogrel use were adjusted for all-cause death; age, COPD, prior coronary artery bypass graft, LVEF, heart rate, rSS, IABP use, and clopidogrel use were adjusted for cardiac death; variables of prior CABG, LVEF, and rSS were adjusted for MI; variables of diabetes, stroke, prior MI, prior CABG, SS, rSS, LAD lesion, IABP use, and GPIIb/IIIa inhibitor use were adjusted for MACCE. In the AMI subpopulation, variables of age, sex, stroke, prior PCI, current smoking, CCr < 60 mL/min, and left main lesion were adjusted for all-cause death; variables of age, prior MI, prior PCI, PAD, left main lesion, CCr < 60 mL/min, and rSS were adjusted for cardiac death; variables of PAD, left main lesion, and rSS were adjusted for MI; variables of LAD lesion, rSS, and GPIIb/IIIa inhibitor use were adjusted for MACCE. CABG: coronary artery bypass graft; COPD: chronic obstructive pulmonary disease; CI: confidence interval; HR: hazard ratio; IABP: intra-aortic balloon counterpulsation; LAD: left anterior descending artery; LVEF: left ventricular ejection function; MACCE: major adverse cardiovascular and cerebrovascular events; MI: myocardial infarction; PAD: peripheral vascular disease.

**Table 2:** Baseline characteristics of patients.

	Overall population			AMI subpopulation			UA subpopulation		
	Discharged with *β*-blockers (*n* = 5,043)	Discharged without *β*-blockers (*n* = 588)	*P* value	Discharged with *β*-blockers (*n* = 1,444)	Discharged without *β*-blockers (*n* = 116)	*P* value	Discharged with *β*-blockers (*n* = 3,599)	Discharged without *β*-blockers (*n* = 472)	*P* value
Clinical characteristics (%)									
Age (years)	58.0 (50.0, 65.0)	59.0 (53.0, 67.0)	0.027	58.0 (50.0, 67.0)	56.0 (48.0, 63.0)	0.009	59.0 (52.0, 66.0)	59.0 (53.0, 67.0)	0.687
Male	3,844 (76.2)	473 (80.4)	0.022	1,220 (84.5)	100 (86.2)	0.621	2,624 (72.9)	373 (79.0)	0.005
BMI (kg/m^2^)	26.0 (23.9, 27.8)	25.4 (23.5, 27.3)	<0.001	26.0 (23.9, 27.8)	25.6 (23.2, 27.1)	0.200	25.9 (23.8, 27.8)	25.3 (23.5, 27.3)	0.002
Diabetes	1,499 (29.7)	127 (21.6)	<0.001	388 (26.9)	21 (18.1)	0.039	1,111 (30.3)	106 (22.5)	<0.001
Hypertension	3,226 (64.0)	356 (60.5)	0.102	832 (57.6)	65 (56.0)	0.740	2,394 (66.5)	291 (61.7)	0.036
Dyslipidemia	3,380 (67.0)	357 (60.7)	0.002	892 (61.8)	60 (51.7)	0.033	2,488 (69.1)	297 (62.9)	0.006
Prior MI	639 (12.7)	72 (12.2)	0.768	92 (6.4)	9 (7.8)	0.559	547 (15.2)	63 (13.3)	0.289
Prior PCI	1,014 (20.1)	125 (21.3)	0.511	247 (17.1)	29 (25.0)	0.032	767 (21.3)	96 (20.3)	0.627
Prior CABG	198 (3.9)	17 (2.9)	0.215	31 (2.1)	2 (1.7)	1.000	167 (4.6)	15 (3.2)	0.148
Stroke	531 (10.5)	61 (10.4)	0.908	125 (8.7)	15 (12.9)	0.121	406 (11.3)	46 (9.7)	0.318
PAD	104 (2.1)	14 (2.4)	0.61	19 (1.3)	3 (2.6)	0.221	85 (2.4)	11 (2.3)	0.966
COPD	112 (2.2)	21 (3.6)	0.041	33 (2.3)	4 (3.4)	0.349	79 (2.2)	17 (3.6)	0.058
Current smoking	2,896 (57.4)	373 (63.4)	0.005	942 (65.2)	91 (78.4)	0.004	1954 (54.3)	282 (59.7)	0.025
Presenting characteristics									
SBP (mmHg)	125 (116, 140)	125 (117, 140)	0.978	120 (110, 130)	120 (105, 130)	0.119	130.0 (120.0, 140.0)	129.0 (120.0, 140.0)	0.892
Heart rate (bpm)	70 (63, 76)	62 (57, 70)	<0.001	70.0 (64.0, 78.0)	61.5 (56.0, 73.8)	<0.001	69.0 (63.0, 76.0)	62.0 (57.0, 69.0)	<0.001
CCr < 60 mL/min (%)	1,939 (38.4)	237 (40.3)	0.382	521 (36.5)	51 (44.0)	0.109	1,412 (39.2)	186 (39.4)	0.942
CK-MB (U/L)	12.0 (9.0, 16.0)	12.0 (9.0, 15.0)	0.209	12.0 (9.0, 17.0)	12.0 (8.0, 15.5)	0.146	11.0 (9.0, 15.0)	12.0 (9.0, 15.0)	0.796
LVEF	63.0 (60.0, 67.0)	64.6 (60.0, 68.0)	0.002	60.0 (55.0, 64.0)	60.0 (55.0, 65.0)	0.659	65.0 (60.2, 68.0)	65.0 (61.0, 68.3)	0.135
40-49%	217 (4.3)	16 (2.7)	0.068	125 (8.7)	10 (8.6)	0.989	92 (2.6)	6 (1.3)	0.087
Procedure characteristics (%)									
SYNTAX score	10 (6, 16)	8 (5, 14)	<0.001	11.0 (7.0, 17.5)	12.0 (7.0, 18.4)	0.435	9.5 (5.0, 16.0)	7.0 (4.3, 12.4)	<0.001
Residual SYNTAX score			0.001			0.178			0.001
0	2,576 (52.7)	350 (60.8)		752 (52.7)	66 (57.4)		1,824 (52.7)	284 (61.6)	
0-8	1,622 (33.2)	157 (27.3)		463 (32.4)	28 (24.3)		1,159 (33.5)	129 (28.0)	
>8	688 (14.1)	69 (12.0)		212 (14.9)	21 (18.3)		476 (13.8)	48 (10.4)	
Left main lesion	122 (2.4)	7 (1.2)	0.058	25 (1.7)	3 (2.6)	0.459	97 (2.7)	4 (0.8)	0.015
LAD	4,588 (91.0)	537 (91.3)	0.779	1,309 (90.7)	103 (88.7)	0.511	3,279 (91.1)	434 (91.9)	0.544
IABP use	49 (1.0)	4 (0.7)	0.489	27 (1.9)	3 (2.6)	0.485	22 (0.6)	1 (0.2)	0.508
IVUS use	276 (5.5)	21 (3.6)	0.051	53 (3.7)	2 (1.7)	0.274	223 (6.2)	19 (4.0)	0.061
GPIIb/IIIa inhibitor use	792 (15.7)	85 (14.5)	0.429	256 (17.7)	21 (18.1)	0.919	536 (14.9)	64 (13.6)	0.442
Medication at discharge									
Aspirin	4,993 (99.0)	568 (96.6)	<0.001	1,437 (99.5)	105 (90.5)	<0.001	3,556 (98.8)	463 (98.1)	0.195
Clopidogrel	4,963 (98.4)	573 (97.4)	0.086	1,427 (98.8)	109 (94.0)	<0.001	3,536 (98.6)	464 (98.3)	0.931
Statin	4,871 (96.6)	547 (93.0)	<0.001	1,395 (96.6)	96 (82.8)	<0.001	3,476 (96.6)	451 (95.6)	0.254
ACEI/ARB	2,742 (54.4)	272 (46.3)	<0.001	1,051 (72.8)	71 (61.2)	0.008	1,691 (47.0)	201 (42.6)	0.071
CCB	2,547 (50.5)	314 (53.4)	0.184	406 (28.1)	33 (28.4)	0.939	2,141 (59.5)	281 (59.5)	0.985

Values are presented as *n* (%), mean ± SD, or median (IQR). ACEI: angiotensin-converting enzyme inhibitors; AMI: acute myocardial infarction; ARB: angiotensin II receptor antagonists; BMI: body mass index; CABG: coronary artery bypass graft; CCB: calcium channel blockers; CCr: creatinine clearance; CK-MB: creatine kinase-MB; COPD: chronic obstructive pulmonary disease; IABP: intra-aortic balloon counterpulsation; IVUS: intravascular ultrasound; LAD: left anterior descending artery; LVEF: left ventricular ejection fraction; PAD: peripheral vascular disease; PCI: percutaneous coronary intervention; SBP: systolic blood pressure; UA: unstable angina.

**Table 3:** 2-year clinical outcomes of the patients.

	Overall population			AMI subpopulation			UA subpopulation		
	Discharged with *β*-blockers (*n* = 5,043)	Discharged without *β*-blockers (*n* = 588)	*P* value	Discharged with *β*-blockers (*n* = 1,444)	Discharged without *β*-blockers (*n* = 116)	*P* value	Discharged with *β*-blockers (*n* = 3,599)	Discharged without *β*-blockers (*n* = 472)	*P* value
Death	47 (0.9)	8 (1.4)	0.317	16 (1.1)	0 (0.0)	0.625	31 (0.9)	8 (1.7)	0.106
Cardiac death	24 (0.5)	3 (0.5)	0.909	10 (0.7)	0 (0.0)	1.000	14 (0.4)	3 (0.6)	0.437
MI	29 (0.6)	5 (0.9)	0.395	8 (0.6)	2 (1.7)	0.167	21 (0.6)	3 (0.6)	0.889
TVR	421 (8.3)	47 (8.0)	0.768	126 (8.7)	9 (7.8)	0.722	295 (8.2)	38 (8.1)	0.913
ST	30 (0.6)	2 (0.3)	0.437	9 (0.6)	0 (0.0)	1.000	21 (0.6)	2 (0.4)	1.000
Stroke	71 (1.4)	8 (1.4)	0.926	14 (1.0)	2 (1.7)	0.336	57 (1.6)	6 (1.3)	0.842
MACCE	538 (10.7)	61 (10.4)	0.827	155 (10.7)	12 (10.3)	1.000	383 (10.6)	49 (10.4)	0.937

Values are presented as *n* (%). ST: stent thrombosis; MACCE: major adverse cardiovascular and cerebrovascular events; MI: myocardial infarction; TVR: unplanned target vessel revascularization.

## Data Availability

The data used to support the findings of this study are restricted by the ethical review board at Fuwai Hospital in order to protect patient's privacy. Data are available from Jin-qing Yuan for researchers who meet the criteria for access to confidential data.
